# Advanced AI-Powered System for Comprehensive Thyroid Cancer Detection and Malignancy Risk Assessment

**DOI:** 10.3390/life16010038

**Published:** 2025-12-26

**Authors:** Noemi Lorenzovici, Horatiu Silaghi, Eva-H. Dulf, Cornelia Braicu, Cristina Alina Silaghi

**Affiliations:** 1 Automation Department, Faculty of Automation and Computer Science, Technical University of Cluj-Napoca, Memorandumului Str. 28, 400014 Cluj-Napoca, Romania; noemi.lorenzovici@gmail.com; 2 Surgical Disciplines, Department 2, Faculty of Nursing and Health Sciences, “Iuliu Hațieganu” University of Medicine and Pharmacy Cluj-Napoca, 8 Victor Babes Street, 400012 Cluj-Napoca, Romania; 3 Physiological Controls Research Center, University Research and Innovation Center, Obuda University, 1034 Budapest, Hungary; 4 Research Center for Functional Genomics, Biomedicine and Translational Medicine, Iuliu Hatieganu University of Medicine and Pharmacy, 400337 Cluj-Napoca, Romania; cornelia.braicu@umfcluj.ro; 5 Department of Endocrinology, “Iuliu Hațieganu” University of Medicine and Pharmacy Cluj-Napoca, 8 Victor Babes Street, 400012 Cluj-Napoca, Romania; alinasilaghi@yahoo.com

**Keywords:** papillary thyroid cancer, medullary thyroid cancer, indeterminate thyroid nodule, molecular diagnosis, computer-aided diagnosis system, deep neural network, convolutional neural network, transfer learning

## Abstract

The thyroid cancer incidence has been continuously rising over the last decades. Recently, intelligent cancer detection software are gaining popularity, due to their high diagnostic accuracy and subsequent direct benefits in avoiding unnecessary surgical interventions. This study introduces a novel hybrid computer-aided diagnosis (CAD) system that combines convolutional neural networks (CNNs) and molecular data analysis to achieve comprehensive and reliable thyroid cancer diagnostics. The system consists of two key modules: The first is a CNN-based model leveraging transfer learning, processes ultrasound images to classify patients as either “healthy” or “with a thyroid nodule.” In cases where a nodule is detected, the second module utilizes molecular data to predict the malignancy risk, providing a probability score for clinical decision support. Different image augmentation techniques (traditional ones as well as novels) were carried out to enhance the robustness of the system. The combination of two independent modules makes it possible to use them decoupled, while used together they provide a powerful, in-depth diagnosis of thyroid cancer. The proposed system demonstrates strong performance: the ultrasound-based CNN module achieves an accuracy of 93.65%, with a sensitivity of 100% and a specificity of 69.23%. For the gene analysis component, the model achieves a training mean squared error (MSE) of 4.24 × 10^−5^ and a testing MSE 6.31 × 10^−3^. These results underscore the system’s competitive performance with existing thyroid cancer detection CAD systems in both diagnostic performance and the depth of insights provided, supporting clinicians in making informed, reliable decisions in thyroid cancer management.

## 1. Introduction

### 1.1. General Context

Thyroid cancer (TC) is the most common endocrine malignancy, showing an increasing incidence over the last decades [1]. In the United States, cancer statistics for the year 2014 indicate that thyroid cancer caused 1950 deaths, and 62,450 new cases were discovered according to [2]. It is projected that TC will be the second most diagnosed cancer in women and the ninth in men by 2030 in the United States [3].

Thyroid cancer development is influenced by a variety of factors, including individual characteristics such as family history and obesity, as well as environmental elements like iodine intake and exposure to carcinogens [4]. Notable risk factors include increased exposure to medical radiation, excessive iodine intake, and a rising incidence of autoimmune thyroiditis [2]. Gender significantly influences TC prevalence, with women being three times more likely than men to develop the disease [5]. Additionally, individuals with a family history of TC are four to ten times more likely to be affected. Genome-wide association studies examining TC and thyroid diseases have highlighted a notable genetic contribution, estimating heritability between 2.5% and 11.3% of cases [6].

There is extensive research on the role of gene expression and mutations in the development and progression of TC [6]. Advances in molecular characterization have identified specific markers detectable in fine-needle aspiration specimens, providing valuable diagnostic tools, particularly for cytologically indeterminate thyroid nodules. Several panels of gene mutational markers (such as Thyroseq v3, Afirma GSC, MPTX) based on molecular classifiers are commercially available. While recent iterations of these panels show improved ability to rule out malignancy, their accuracy in confirming malignancy still requires refinement [7].

Despite advancements in molecular research, thyroid ultrasonography (US) remains the primary tool for detecting TC, typically presenting as thyroid nodules. Ultrasonography not only identifies nodule size and characteristics but also detects cervical lymph node metastasis and guides fine-needle aspiration. However, its limitations include variability in operator experience, equipment performance, and the lack of standardized reporting [8]. To mitigate these issues, ultrasonography risk stratification systems, such as TIRADS, have been introduced to assess malignancy risk [9]. While promising, most TIRADS validation studies are retrospective with inconsistent results, limiting their clinical utility [8].

Recent work in multimodal medical artificial intelligence further highlights the importance of integrating heterogeneous data sources—such as imaging, molecular, and clinical information—within unified AI frameworks, while carefully assessing robustness and generalization across modalities. In particular, multimodal medical AI systems combining imaging and molecular data have been shown to improve diagnostic performance when each modality is appropriately validated and interpreted within a clinical decision-support context [10]. According to [11], nearly 30% of surgically resected indeterminate nodules are benign, highlighting the challenge of avoiding unnecessary surgeries. Combining genetic analysis with US can improve TC diagnosis and clinical management, in assessing whether to apply or not surgical treatment and subsequently in selecting the optimal surgical options (lobectomy versus initial total thyroidectomy and central neck dissection) in indeterminate thyroid nodules. Since early detection is crucial for improving outcomes, intelligent cancer detection systems are gaining popularity, due to their high accuracies and their ability to detect features that the human eye cannot see on an ultrasound image.

This study introduces an innovative computer-aided diagnosis (CAD) system that combines ultrasound image analysis with molecular data to enhance thyroid cancer diagnostics. The system operates in two distinct phases: Initially, it classifies ultrasound images in BMP format as either “healthy” or “nodule present.” If a nodule is detected, the second phase employs a genomic analysis model, leveraging data on gene mutations and miRNA expression levels in tumoral versus normal tissue, to estimate the malignancy probability. A novel synthetic dataset was specifically developed to train this genomic model, improving its diagnostic performance.

The study’s primary contributions lie in two key areas. Firstly, it introduces an advanced diagnostic approach for thyroid cancer, using a significantly expanded set of genomic predictors. Unlike prior research, which typically focuses on fewer than 10 genomic factors, this work incorporates 46 predictors, including the most frequently mutated genes and altered miRNA patterns in thyroid cancer, based on data from The Cancer Omics Atlas [12]. The integration of these predictors into a synthetic dataset and their application in a CAD system represent a novel advancement in the field. Secondly, the system achieves a unique integration of molecular data analysis and robust ultrasound image processing, traditionally treated as separate diagnostic methods. The success of the image processing tool consists of the intensive study of efficiency of five different convolutional neural network architectures as well as different image augmentation techniques and their effect on the accuracy of the model. By combining these approaches, the CAD system provides a more comprehensive and reliable diagnostic tool for thyroid cancer. This integrated framework ensures a synergistic analysis that strengthens diagnostic accuracy compared to stand-alone tools.

The methodologies include convolutional neural networks (CNNs) for ultrasound image processing and deep neural networks (DNNs) for genomic data analysis. MATLAB 2022b was chosen as the development environment for the diagnostic prediction system. After the neural network models were designed and trained, they were integrated into a standalone desktop application capable of diagnosing thyroid cancer in two stages, using both echography images and genomic data.

### 1.2. Literature Review

As mentioned above, genetics play an essential role in TC. TC is a relatively low mutational burden tumor, with identifiable driver mutations in more than 90% of cases. The two main pathways involved in the pathogenesis of TC are mitogen-activated protein kinase (MAPK) and phosphatidylinositol-3 kinase (PI3K)/AKT signaling pathways. Approximately 45% of PTC carry point mutations in the BRAF oncogene, activating the MAPK pathway. Papillary thyroid cancer (PTC) with the most common V600E mutation seems to have a more aggressive long-term disease progression. In addition, point mutations of RAS genes occur in 10–20% of PTC, usually in the follicular variant of PTC. The most frequent chromosomal rearrangements are the rearranged during transfection (RET)/PTC1 fusions that appear in 20% of the PTCs. Also, NTRK1 translocations are present in 5–8% of the PTCs [13]. The regulation of miRNAs also tends to be involved in the pathophysiology of PTC as, for example, the downregulation of miRNA-369-3p and the consequent upregulation of its target TSPAN13 [14,15] studies the relationship between the gene expressions of three miRNAs (namely mir-146b, miR-221, and miR-222) and the development of TC. It concludes that these miRNAs are associated with higher cancer aggressiveness in the case of the PTC. The three miRNAs could also serve as potential prognostic biomarkers in PTC [16].

In follicular thyroid cancer (FTC), the critical activation of the PI3K/AKT pathway is triggered by activating mutations of RAS in nearly 50% of cases, involving the KRAS, HRAS, or NRAS oncogenes. Also, FTC may carry a PAX8/PPARγ1 fusion in 30–40% of FTC [17]. Conversely, BRAF and TERT mutations are found more frequently in ATC than in DTC [18].

Another type of TC is medullary thyroid carcinoma (MTC) a neuroendocrine cancer that derives from the parafollicular C-cells and accounts for approximately 3–5% of all TC. MTC shows a potentially aggressive behaviour with early lymph node metastasis. Activating mutations of the RET proto-oncogene are responsible for almost all cases of hereditary or familial MTC and 40–50% of “sporadic” MTC. Also, activating mutations of the RAS gene, mainly HRAS and KRAS, are harboured in approximately 13% of “sporadic” MTC [19,20].

CAD systems incorporating gene markers have gained significant attention for their enhanced performance and reliability in medical diagnostics [21,22]. Algorithmic analysis of cancer-related molecular data has long been employed to identify biologically meaningful patterns associated with tumorigenesis, such as conserved sequence motifs in tumor suppressor proteins, providing early bioinformatics foundations for the computational extraction of informative molecular features in oncology [23]. Furthermore, in [24] study, regression analysis combined with a fuzzy model is used to select gene markers in diagnosing ovarian cancer and then classify the type of cancer. In the context of TC, reference [25] developed a system based on artificial neural networks that uses genomic information to classify patients into low- or high-risk categories, achieving an accuracy of 77.5% and a specificity of 86%. Similarly, reference [26] presents an intelligent system based on deep neural networks to analyse ncRNAs (non-coding RNAs) in a complex gene regulatory network to predict the presence of TC. Expanding on these approaches, reference [27] proposed a deep learning model that predicts specific gene mutations from image inputs with an accuracy of 95.2% for BRAF and RAS mutations.

Integrative approaches have also shown promise in enhancing TC diagnostics. The article [28] utilized a Bayesian network to combine molecular and clinical data, demonstrating the added predictive value of incorporating clinical features alongside molecular markers for malignancy risk assessment. Another study [29] identifies key RNA transcripts from The Cancer Genome Atlas dataset and develops machine learning models to classify thyroid carcinoma stages, achieving robust accuracy in distinguishing early and late-stage samples as well as cancerous and normal tissues. Furthermore, the study [30] develops and evaluates an mRNA-based molecular classifier trained on fine-needle aspirates to distinguish benign from malignant thyroid nodules, demonstrating high predictive accuracy and robustness to RNA degradation and cellular heterogeneity in clinical settings.

Recent literature increasingly supports synthetic genomic data as a practical enabler for model development and method benchmarking, provided that reliability is established through transparent evaluation of fidelity, downstream analytical utility, and privacy risk [31]. For example, diffusion-based approaches have been proposed to generate synthetic human genotypes that reproduce key population-genetic structures, while remaining useful for downstream analyses when compared against real genotypes [32]. In parallel, studies investigating artificial genomes for GWAS-style workflows further emphasize that “clinical reliability” in the context of synthetic genomics should be operationalized as preservation of task-relevant signal rather than superficial similarity alone [33]. Moreover, benchmarking work highlights that utility must be interpreted alongside privacy testing (e.g., membership inference vulnerability), motivating careful reporting of both utility and privacy when synthetic genomic datasets are used for training predictive models [34].

Within oncology, synthetic data generation has also been explored as a way to reproduce complex genomic alteration patterns and augment limited cohorts. Synthetic cancer genome generation methods have been shown to reproduce somatic mutation profiles and other genomic alterations across cancer types, supporting the feasibility of generating genomically realistic training data when appropriately validated [35]. A focused review of synthetic data in genomic cancer medicine further indicates that evaluation-centered reporting is central to ensuring that synthetic genomic data are used responsibly and interpreted correctly [36].

Large-scale genomic analyses have shown that thyroid cancer is driven by multiple recurrent genetic and regulatory alterations rather than isolated mutations, motivating the use of multi-gene molecular representations [37]. In addition, both multigene expression–based molecular classifiers and miRNA dysregulation—particularly involving miR-146b and the miR-221/222 family—have been shown to improve diagnostic and prognostic assessment in thyroid nodules, supporting the inclusion of both mutation-driven genes and regulatory miRNAs as predictive features [30,38].

Building on the TC mutational landscape, the current work integrates 42 of the most frequently mutated genes and four overexpressed miRNAs as predictors in the development of an intelligent CAD system, aiming to advance the accuracy and reliability of TC diagnostics.

Image processing is a crucial component of computer-aided diagnosis systems, encompassing tasks such as pre-processing, feature extraction, segmentation, and classification. A notable example is [39]’s mark-guided deep network model, which significantly enhances ultrasound-based thyroid nodule segmentation, aiding clinical diagnosis and treatment. Image classification is another common CAD application, distinguishing benign from malignant nodules. For instance, reference [40]’s software achieves precision rates of 96.7% and 95.3%, while [41] study reports 97.5% accuracy using fuzzy logic and support vector machines for malignant nodule detection.

Convolutional neural networks are specialized for high-accuracy image processing, making them widely used in medical imaging to delineate tumour contours, classify images as healthy or cancerous, and identify cancer types, including thyroid cancer [42,43]. Transfer learning, which fine-tunes pre-trained CNNs for specific tasks, is universally practiced to reduce computational demand and enhance accuracy [44]. Additionally, comparing multiple pretrained deep CNN architectures to detect various cancer types (lung, breast, thyroid, etc.) or optimize feature extraction has also demonstrated effectiveness [45,46].

In the literature of thyroid cancer detection using transfer learning and CNNs, similar to this work, reference [47] compares four CNN models, among which the best performing one achieves an accuracy of 75%, sensitivity of 84.9% and specificity of 69.0%. Additionally, reference [48] study evaluates various transfer learning models, including DenseNet169, ResNet101, and EfficientNet variants, for classifying thyroid nodules on ultrasound images, identifying DenseNet169 as the most accurate model with 95.96% accuracy. The research underscores the potential of AI in enhancing the precision and reliability of thyroid disease diagnosis compared to traditional methods.

Transfer learning is also used in the novel methodology presented by [49], combining U-Net and VGG16 architectures for segmenting thyroid nodules in infrared thermal images. According to the authors, the pre-trained VGG16 layers were used for feature extraction, which were then processed by a U-Net-based decoder to enhance segmentation accuracy. A key innovation of this approach lies in incorporating feature engineering to improve segmentation performance, even with a limited dataset. The experimental results demonstrated that integrating radiomics-based feature engineering significantly improved the Dice coefficient, highlighting the potential of CNN-based models in refining thyroid nodule segmentation through thermal imaging.

The [50] study develops an efficient CAD system for thyroid tumour characterization using ultrasound images, employing Edge Preserving Smoothing filters and a ResNet50-based segmentation model for pre-processing. It evaluates 15 pre-trained deep learning models as feature extractors, namely AlexNet, VGG16, VGG19, Darknet19, Darknet53, GoogleNet, DenseNet201, ResNet18, ResNet50, ResNet101, EfficientNetb0, NasNetMobile, MobileNet, SqueezeNet, and ShuffleNet and identifies ResNet50 as the optimal model, achieving a classification accuracy of 99.5% with a PCA-SVM classifier to distinguish between benign and malignant tumours.

Highlighting the benefits of transfer learning, research [51] introduces an automated approach for thyroid lesion localization and classification in ultrasound images by utilizing FCN-AlexNet for image segmentation and lesion localization, followed by AlexNet for classifying the localized regions as benign or malignant. By integrating transfer learning to mitigate training data limitations, the proposed method achieves an IoU of 0.82 for lesion localization and diagnostic metrics of 90.8% accuracy, 91.4% sensitivity, 90.4% specificity, and an AUC of 0.952, outperforming other methods on the same dataset.

Several recent studies support the continued relevance of VGG-based architectures in thyroid ultrasound analysis, particularly within transfer learning frameworks. In a comparative evaluation of pretrained deep CNNs for thyroid nodule classification, VGG-19 was retrained alongside more recent architectures such as InceptionV3 and ResNet101, demonstrating diagnostic performance comparable to deeper and more complex models, thereby confirming its suitability for ultrasound-based malignancy assessment despite architectural simplicity [52]. Complementing this finding, in [53] a dedicated VGG-19–based model (BETNET) was specifically designed for thyroid nodule classification, where fine-tuning of pretrained VGG layers enabled effective extraction of discriminative ultrasound features, reinforcing the adaptability of VGG-19 in medical imaging tasks. Furthermore, a large multicohort study involving thousands of thyroid ultrasound images evaluated VGG-16, VGG-19, and ResNet-based models, reporting that VGG-16 achieved diagnostic performance comparable to that of experienced radiologists, underscoring the robustness and clinical relevance of VGG architectures when applied through transfer learning [54]. Collectively, these studies demonstrate that VGG-16 and VGG-19 remain strong and well-validated baseline models for thyroid cancer detection, particularly in settings where interpretability, training stability, and reliable feature extraction from ultrasound images are critical.

Within this context, in this study the selection of five CNN architectures—AlexNet, VGG-16, VGG-19, DarkNet-19, and ResNet-50—was motivated by their complementary architectural characteristics, proven effectiveness in medical imaging, and frequent use as benchmark models in thyroid ultrasound analysis. AlexNet represents an early deep CNN architecture that remains relevant due to its relatively low computational complexity and robust feature extraction capabilities, making it suitable for datasets of moderate size and a common baseline in CAD systems. In comparison, VGG-16 and VGG-19 leverage deeper stacks of small (3 × 3) convolutional filters that have been shown to capture fine-grained textural and morphological features in ultrasound images; VGG-based transfer learning has been successfully applied to thyroid nodule classification with notable performance [49,50,52,53,54]. DarkNet-19, originally developed for real-time object detection, offers a favorable balance between depth and computational efficiency and has demonstrated competitive performance in medical image classification problems, including thyroid tumor characterization [39]. ResNet-50 introduces residual connections that address vanishing gradient issues in deeper networks and has consistently achieved state-of-the-art results in thyroid ultrasound CAD systems, often outperforming earlier architectures in large-scale comparative studies [30,39,52,54]. By evaluating these five architectures under identical experimental conditions, the present study provides a structured comparison between classical and more modern CNN designs, allowing performance differences to be attributed to architectural properties rather than data or training discrepancies.

## 2. Materials and Methods

### 2.1. Ultrasound Images

#### 2.1.1. The Data

**Figure 1:**
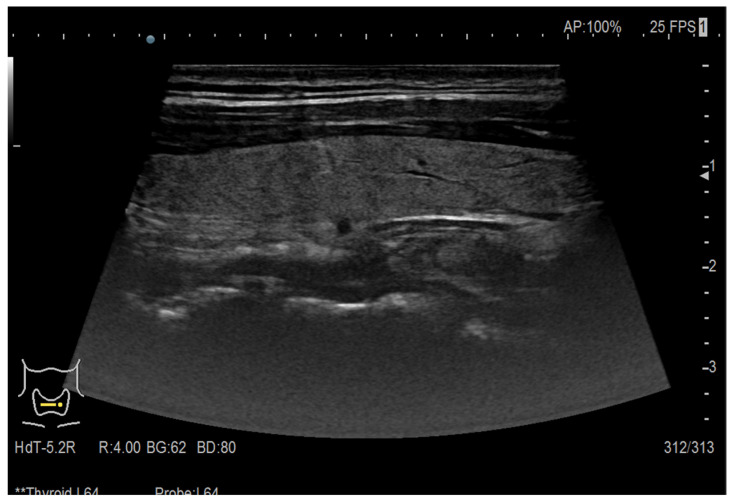
Ultrasound image of a healthy thyroid.

**Figure 2:**
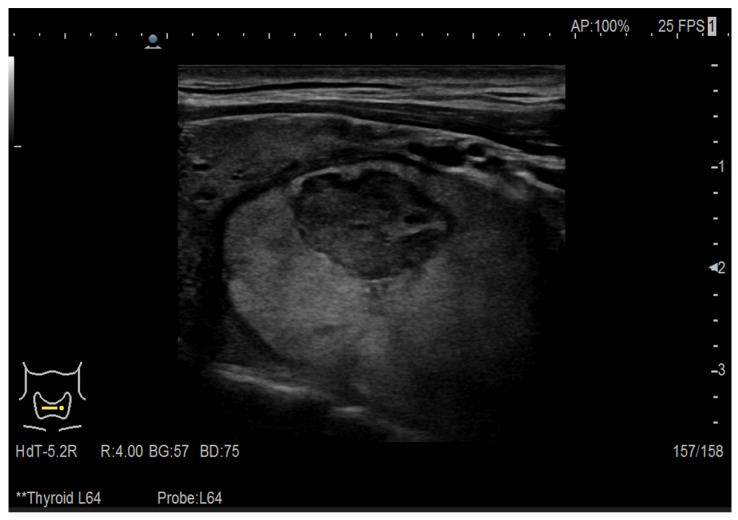
Ultrasound image of a thyroid with nodule.

The data used to develop the CAD system for thyroid nodule detection consisted of 420 images from multiple ultrasound machines, each image obtained from a different patient to ensure dataset heterogeneity and prevent redundancy. The cohort included 278 female and 142 male patients, aged 36–71 years. Among these, 225 were ultrasound images of thyroids with nodules (malignant and benign) and 195 images of healthy thyroids, both class labels obtained from the medical expert among the authors, after performing fine needle aspiration (FNA) cytology. In Figure 1 the echography of a healthy thyroid is presented, while Figure 2 shows a patient with a nodule in the thyroid gland.

#### 2.1.2. Data Preprocessing

To ensure that only relevant anatomical details were retained while eliminating non-diagnostic information, a preprocessing pipeline was applied to all ultrasound images before model training. Given the limited dataset size, it was crucial to remove artifacts while preserving the essential features required for classification. Each image underwent an automated artifact removal process, designed to eliminate extraneous information such as patient identifiers, examination dates, and machine-generated annotations (Figure 1 and Figure 2). These artifacts, commonly present in ultrasound scans, introduce noise into the dataset and may lead to unintended biases during model training.

**Figure 3:**
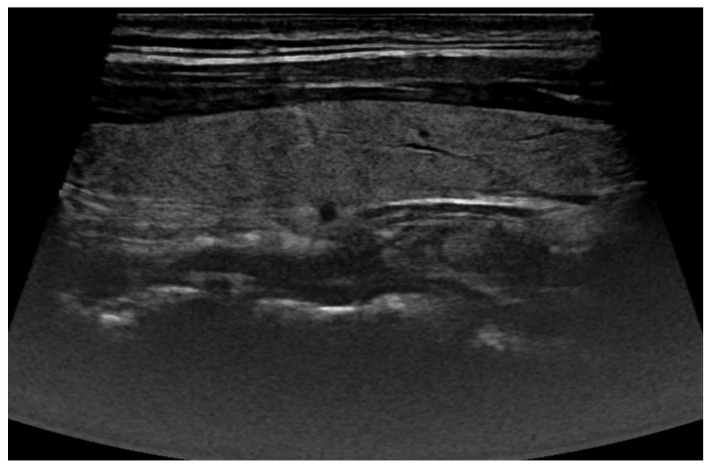
Ultrasound image after preprocessing pipeline.

To achieve this, a cropping and resizing function was implemented. The function processed each image by identifying the central anatomical region and cropping it into a rectangular shape, ensuring that the diagnostically relevant portions remained intact. This approach was chosen to maintain the spatial integrity of thyroid structures while completely removing extraneous information from the outer regions. Following artifact removal, images were rescaled to a uniform resolution to ensure consistency across the dataset. Standardized image dimensions are essential for deep learning models, as they facilitate efficient feature extraction and improve network generalization. Figure 3 illustrates one resulting US image from the preprocessing pipeline.

By applying this structured preprocessing pipeline, the dataset was refined to focus solely on relevant echographic features, reducing the risk of model misinterpretation, the influence of machine-specific artifacts, operator variability, and other non-diagnostic variations and enhancing robustness across diverse ultrasound scans.

#### 2.1.3. Data Augmentation

To address the challenge of limited data and improve model performance, four data augmentation techniques were applied, each increasing the dataset size by 25%, resulting in a total of 840 images (450 thyroid nodule images and 390 normal thyroid images). The chosen techniques included random rotation (±20°) and translation of the images along the X and Y axes (±15 pixels), contrast enhancement using contrast-limited adaptive histogram equalization (CLAHE) with a tile size of 8 × 8 able to grasp fine anatomical features, and Gaussian noise with sigma values ranging from 1 to 3, added to 20% of the augmented images to mitigate overfitting. The chosen augmentation techniques were specifically selected to simulate realistic ultrasound variations. Random rotation and translation mimic differences in probe orientation and patient positioning, CLAHE compensates for variability in imaging settings and tissue echogenicity, and Gaussian noise models speckle inherent to ultrasound imaging. Together, these augmentations were meant to enhance the model’s ability to generalize across images acquired under different clinical conditions, thereby reducing sensitivity to noise and acquisition variability.

To prevent data leakage, the dataset was split at the patient level, ensuring that all images (original and augmented) from the same patient were assigned to the same subset. Additionally, augmented images were assigned to the same subset as their original counterparts, ensuring that no image or its augmented version appeared in different subsets. This maintained a clear separation between the training, validation, and test sets, preserving the integrity of model evaluation. The dataset was divided into 60% for training, 20% for validation, and 20% for testing.

#### 2.1.4. Model Architecture

The method of transfer learning was employed to train five different models and compare their performance in terms of accuracy, sensitivity, specificity, and training time. The chosen architectures were: ALEXNET, DARKNET-19, VGG-16, VGG-19, and RESNET-50 due to their wide usage in the field of thyroid cancer detection. The pretrained models were fine-tuned using identical training configurations: stochastic gradient descent with momentum (momentum = 0.9) as the optimization algorithm, an initial learning rate of 10^−4^, a maximum of 6 epochs, and a minibatch size of 10, which was selected to accommodate the relatively small dataset and to improve convergence stability during training. Binary cross-entropy was used as the loss function. No explicit class balancing techniques (e.g., weighted loss, oversampling, or undersampling) were applied. However, the dataset after augmentation included 450 thyroid nodule images and 390 healthy thyroid images, which was considered sufficiently balanced for training.

The dataset was split into training, validation, and hold-out test subsets, with 60% of images used for training, 20% for validation, and 20% reserved for final testing. During training, model performance was monitored on the validation set to ensure convergence and prevent overfitting. Final evaluation on the test set employed standard metrics including accuracy, sensitivity, and specificity, providing a comprehensive assessment of the models’ ability to correctly detect thyroid nodules and identify healthy cases.

To assess the impact of data augmentation, ALEXNET was first trained on five different datasets: one without augmentation and four incorporating a combination of augmentation techniques (rotation, translation, CLAHE, and Gaussian noise). The ALEXNET architecture was proposed to test the significance of each augmentation technique due to its relatively low level of complexity compared to the other network architectures. This experiment helped evaluate the contribution of each augmentation method to the model’s performance before applying them collectively to the final dataset for training all five CNN architectures.

The best-performing neural network, identified based on accuracy, sensitivity, specificity, and training time, was subsequently exported for use in the intelligent diagnosis system.

### 2.2. Gene Analysis

The second aim of the study was to define the risk of malignancy in case of a detected thyroid nodule, using molecular data. The model developed for this task aimed at giving a diagnosis in terms of a percentage concerning the probability of having TC from qualitative data. This qualitative dataset consisted of labels regarding genomic information. Since a synthetic dataset was used for training the model, the presented method offers a proof of concept (POC) regarding the use of these genomic predictors in the detection of thyroid cancer rather than attempting to exactly replicate patient-level genomic dependencies.

#### 2.2.1. Dataset Construction

**Figure 4:**
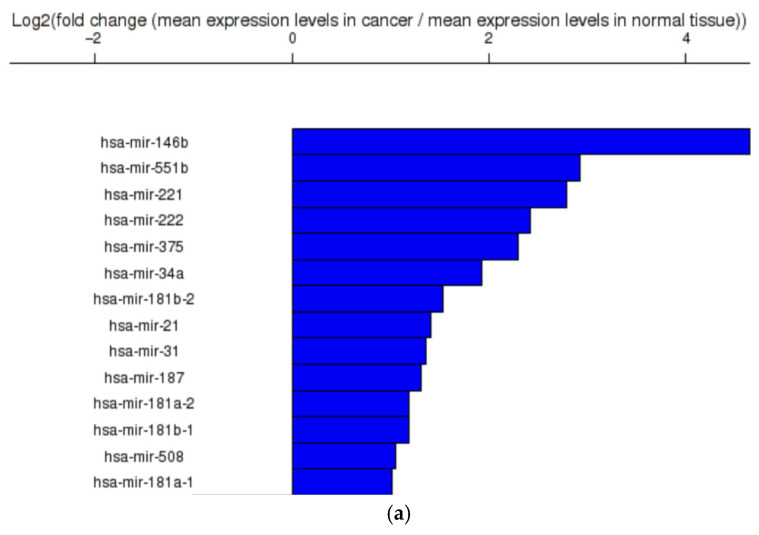

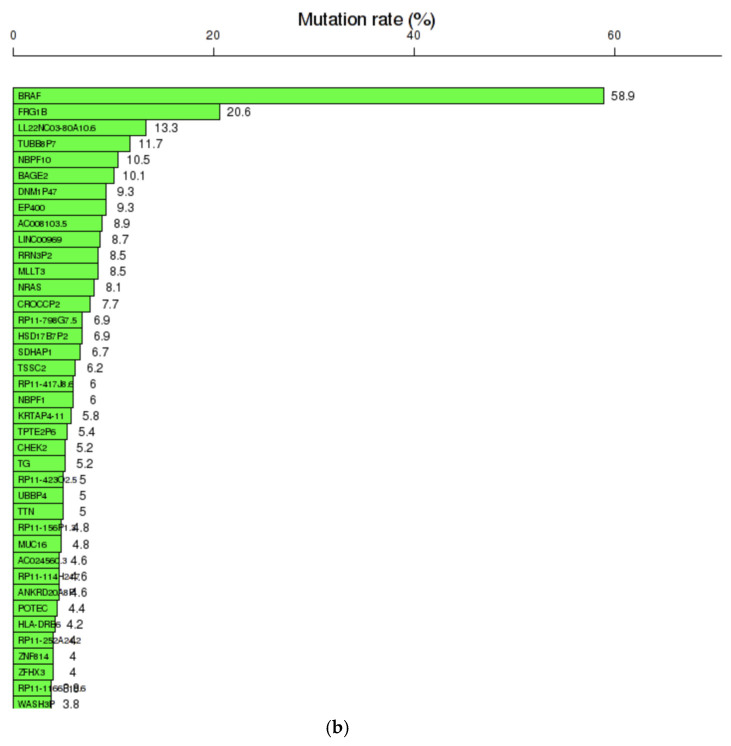
(**a**) Top altered miRNAs in TC (adapted from Figure 4D in [12]); (**b**) Top frequented mutated genes in TC, data downloaded from TCOA (adapted from Figure 4A in [12]).

In order to construct the novel synthetic dataset of genomic predictors, data related to the main 4 altered miRNAs in TC (Figure 4a) and the main frequented mutated genes (Figure 4b) was downloaded from TCOA, an online bioinformatic tool developed based on The Cancer Genome Atlas (TCGA) dataset [41]. The aim of this task is to create a set of artificial patient records that are very diverse and closely represent the main types of this disease. It was used a generative adversarial network (GAN) to recreate artificial versions of the complex genomic sequences of the patients. The used conditional GAN consisted of two networks, one generator and one discriminator, each of theme trained adversarially. The generator creates artificial results for the discriminator, used along with real data as inputs for the discriminator, which has to identify which outputs are real and which are synthetic, to obtain generated data with the same distribution as the real one. Based on [12,55], it was implemented a conditional GAN with gradient penalty extracted from top altered miRNAs in TC and top frequented mutated genes in TC, data downloaded from TCOA. This ensures complex distribution of the results. Distribution, correlation, and principal component analysis evaluation were then assessed on all data types.

The innovative synthetic dataset consisted of an excel table having 590 virtual patients’ data, and each row corresponded to one virtual patient. There were 47 columns in the table reflecting the 46 predictors and the response variable.

The first 42 columns contained the top frequented mutated genes in TC: BRAF, FRG1B, LL22NC03-80A10.6, TUBB8P7, NBPF10, BAGE2, DNM1P47, EP400, AC008103.5, LINC00969, RRN3P2, MLLT3, NRAs, CROCCP2, RP11-796G7.5, HSD17B7P2, SDHAP1, TSSC2, RP11-417J8.6, NBPF1, KRTAP4-11, TPTE2P6, CHEK2, TG, RP11-423O, UBBP4, TTN, RP11-156P1.3, MUC16, RP11-114H24, POTEC, HLA-DRB6, ZNF814, ZFHX3, WASH3P, SNHG14, NCOA6, HRAS, PRSS3P2, ZNF733P, MT-ND5, MUC4. The next four columns contained top 4 overexpressed miRNAs in TC, according to TCOA database: hsa-mir-146b, hsa-mir-551b, hsa-mir-221, hsa-mir-222. If the mutations were present in the patient’s genome, then the column contained the label ‘1’, otherwise ‘0’. Similarly, if the level of selected miRNA was altered (considering as cut-of value a fold-change (FC) of expression levels in cancer versus normal tissue of ±2 and a *p*-value ≤ 0.05 (Figure 4) the corresponding column was labelled with ‘1’, otherwise ‘0’. The final column contained the binary response variable, ‘1’ for a malignant tumour and ‘0’ for benign cases.

To reduce the impact of implausible feature combinations, the generation process was guided by mutation frequency statistics reported in TCGA-derived resources and refined through expert consultation, ensuring that the resulting synthetic patient profiles remained biologically plausible. Given the binary nature of the predictors and the exploratory scope of the study, explicit feature decorrelation was not enforced. Instead, robustness to potential multicollinearity and synthetic noise was addressed at the modelling level through normalization of feature weights, bounded input representations, and evaluation of generalization performance using independent training and testing subsets. This design choice reflects the intended use of the genomic classifier as a complementary decision-support tool rather than a definitive molecular diagnostic assay.

Following the creation of the synthetic dataset, a deep neural network model was developed to predict thyroid cancer based on genomic data. The input to the model was structured as a 46-dimensional column vector, representing the selected predictors (the top 42 most frequently mutated genes and the top 4 overexpressed miRNAs associated with thyroid cancer), while the output was a scalar value providing the probability of malignancy.

Each predictor was assigned a weight reflecting its relative contribution to malignancy risk (the response variable). These weights were determined using a two-fold approach: (1) expert judgment regarding the clinical relevance of each gene mutation and expression level, and (2) mutation rates (Figure 4b) for the top 42 most frequently mutated genes, as higher mutation rates are strongly correlated with increased cancer risk [44]. To ensure consistency, predictor weights were normalized such that their sum equaled 100%. The binary response variable, indicating the malignancy status of the thyroid nodule (benign or malignant), was derived through consultation with the gene analysis author of the study.

#### 2.2.2. Model Architecture

Building on the findings of [56], which demonstrated the effectiveness of a feedforward deep neural network with backpropagation consisting of five hidden layers and 20 neurons per layer for high-accuracy data processing, this architecture was selected for the cancer diagnosis software, using the Levenberg–Marquardt algorithm, which operates in batch mode using the entire training set per iteration. A maximum of 1000 training iterations was set. Model evaluation was performed using the mean squared error (MSE) as the performance metric, reflecting the regression nature of the task. The dataset was divided into training (60%), validation (20%), and testing (20%) subsets. Model performance was monitored on the validation set to prevent overfitting, and training was stopped when validation mean squared error plateaued. The final evaluation was performed on the hold-out test set, using MSE as the primary performance metric. This approach ensures robust estimation of the model’s ability to predict malignancy probability from genomic features and allows reproducibility of the training and evaluation process. Both the image processing and the regression models were trained on a computer having an i7 9700K, 4.7 GHz Turbo Boost CPU and 16 GB DDR4 memory, ensuring reproducibility of the training and evaluation process.

## 3. Results and Discussions

### 3.1. Thyroid Nodule Diagnosis Based on Ultrasound Images: Binary Classification Problem Solved Using Convolutional Neural Networks

The impact of data augmentation on model performance was first analysed using ALEXNET. Table 1 summarizes the results, showing that all augmentation techniques improved model accuracy, supporting their combined use in preparing the final dataset for training the five CNN architectures. The combination of the 4 augmentation techniques increased the model performance by more than 12%.

**Figure 5:**
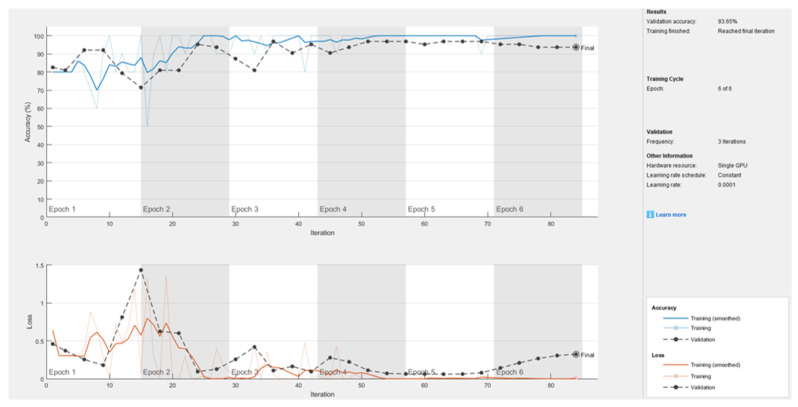
Training results of VGG-19 obtained using transfer learning.

Following the augmentation analysis, the five pretrained CNNs (ALEXNET, DARKNET-19, VGG-16, VGG-19, and RESNET-50) were fine-tuned on the dataset, and their performance metrics were compared. The VGG-19 model emerged as the best-performing architecture, achieving an accuracy of 93.65% and a sensitivity of 100%, correctly identifying all patients with thyroid nodules in the validation set. Figure 5 presents the training progress of the CNN model across six epochs. The top plot illustrates the accuracy curves, where both training and validation accuracy increase steadily and stabilize, reaching a final validation accuracy of 93.65%. The bottom plot depicts the loss curves, showing a consistent decrease in training and validation loss, indicating effective learning and model convergence. The alignment between training and validation curves confirms that the model generalizes well, with no evidence of overfitting.

Comparing the best performing VGG-19 model with the other 4 architectures, the training times were consistent across all models, remaining under 20 min, indicating efficient fine-tuning. While VGG-16 demonstrated the highest specificity (92.3%), its sensitivity was the lowest at 88%. Given the clinical importance of accurate cancer detection, sensitivity was prioritized over specificity, as it reflects the model’s ability to identify true positives. Overall, the VGG-19 model balanced high accuracy and sensitivity, making it the most suitable candidate for thyroid nodule detection. A summary of the performance metrics for all CNNs is presented in Table 2.

### 3.2. Thyroid Cancer Diagnosis Based on Genes and Gene Mutations: Regression Problem Solved with Deep Neural Network

After training the DNN, the model achieved a training MSE of 4.24 × 10^−5^ and a testing MSE of 6.31 × 10^−3^, demonstrating high precision in modelling malignancy risk based on molecular data.

Comparing these minimized MSE values to those reported in similar DNN-based cancer detection systems underscores the superior performance of this approach. For instance, in the study by [46] a similar approach is presented to identify breast cancer using neural networks, obtaining a final MSE equal to 0.043, significantly higher than the results presented in this current paper. Similar trends are observed in other CAD systems, such as the lung cancer detection software by [47] with a minimum MSE of 0.0942. Moreover, in a recent papillary thyroid carcinoma diagnosis study [48] incorporating imprinted gene detection, the predictive model demonstrated strong classification performance, achieving a mean absolute error (MAE) of 0.033 and a mean squared error (MSE) of 0.002. While this approach shows promise in diagnostic accuracy, the deep learning methodology employed in the present study yields an even lower testing MSE, indicating improved precision in malignancy risk assessment based on molecular data. These results reinforce the robustness of the proposed method and its potential to contribute to more accurate and reliable cancer diagnosis.

### 3.3. Integration of the Two Diagnosis Models

**Figure 6:**
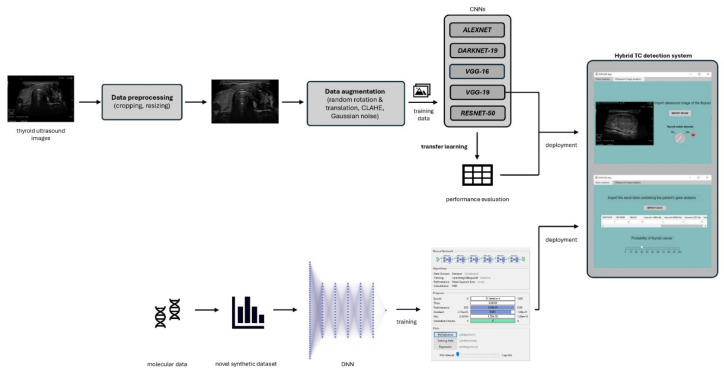
Architecture of the novel hybrid TC detection system.

The two diagnostic models were integrated into a single software application to provide a comprehensive and reliable tool for thyroid cancer diagnosis. The software features two main modules: the first one leveraging a VGG-19-based architecture to classify thyroid nodules from ultrasound images, and the second one for assessing the malignancy risk of a detected nodule, providing a probability score for cancer diagnosis from molecular data (Figure 6). Designed for flexibility, the modules can be utilized independently or jointly, depending on clinical needs and data availability. The software was developed using MATLAB Application Designer and deployed as a standalone desktop application.

From a clinical workflow perspective, thyroid ultrasound is typically used to characterize nodules and guide decisions regarding FNA and follow-up, often within standardized risk stratification frameworks. In this context, the proposed two-module system can be viewed as a decision-support pipeline: an initial ultrasound-based module that screens for nodule presence, followed—when a nodule is detected—by a molecular-risk module that estimates malignancy probability to support management decisions, particularly in indeterminate or borderline scenarios. Importantly, translation to routine practice requires external validation on independent multi-institutional cohorts, with explicit testing across different ultrasound machines, acquisition settings, and operators, as well as calibration analyses that assess whether predicted probabilities remain reliable across sites. Accordingly, external cross-institutional evaluation is a necessary next step before clinical adoption.

Recent studies increasingly evaluate AI not only as an image classifier but as a tool that can shift clinical workflows around FNA, potentially improving efficiency and cost/benefit. For example, a large retrospective and prospective multicentre study developed deep learning models for thyroid FNA cytopathology classification using whole-slide images, positioning AI as an assistive system in settings with limited cytopathology resources [57]. In parallel, clinical evaluations of FDA-cleared ultrasound decision-support tools have examined whether AI recommendations could reduce potentially avoidable FNAs while maintaining acceptable sensitivity for malignancy, directly linking model output to biopsy decision points, presenting a sensitivity of 85.7% and a specificity of 53.3% [58].

A growing body of recent work also supports the value of multimodal approaches in thyroid cancer diagnostics, particularly those combining ultrasound imaging with molecular testing or additional clinical data to improve risk stratification. Several recent studies have demonstrated that integrating ultrasound-based AI with molecular markers can enhance diagnostic precision, especially for cytologically indeterminate nodules, by improving positive predictive value while preserving sensitivity. The metrics presented in the paper are comparable to our results: the proposed model demonstrated a sensitivity of 94.6% and specificity of 70.3% [59]. Compared with these approaches, the proposed system adopts a modular two-stage design in which ultrasound-based screening (healthy versus nodule present) precedes molecular malignancy risk estimation. While many multimodal studies focus on benign versus malignant classification within preselected nodule cohorts, the present framework emphasizes flexible integration within the broader diagnostic pathway. Consequently, direct metric-to-metric comparison should be interpreted cautiously; nevertheless, the reported results support the feasibility and clinical relevance of a multimodal CAD system aligned with current diagnostic workflows.

## 4. Conclusions

In conclusion, this study presents a novel hybrid CAD system that integrates convolutional neural networks and molecular data analysis to enhance the accuracy and reliability of thyroid cancer diagnostics. The system comprises two modules: the first utilizes CNNs with transfer learning to classify ultrasound images, while the second leverages genomic data to assess malignancy risk. This dual-module design offers flexibility, allowing independent use or combined operation for more comprehensive diagnostics.

Among the five CNN architectures evaluated (ALEXNET, DARKNET-19, VGG-16, VGG-19, RESNET-50), VGG-19 demonstrated the highest performance, achieving 93.65% accuracy, 100% sensitivity, and 69.23% specificity. The model’s exceptional sensitivity highlights its potential for detecting thyroid nodules from ultrasound images, positioning it as a valuable tool for clinical application. Comparisons with existing CAD systems (in work [37] an accuracy of 75% is presented, while in work [40] an accuracy of 90.8% is achieved for TC detection) reveal that while accuracies exceeding 95% are rare, the achieved accuracy of 93.65% falls within the upper performance range, underscoring the system’s efficacy.

The second module, a deep neural network trained on genomic data, achieved a training mean squared error of 4.24 × 10^−5^ and a testing MSE 6.31 × 10^−3^, reflecting the robustness of the genomic analysis component. This performance indicates low prediction error on the present dataset and supports the robustness of the proposed genomic analysis component; however, direct quantitative comparison across studies is limited by differences in data sources, feature selection, and validation design.

The innovation of this work lies in the integration of synthetic genomic data with real ultrasound images, addressing the limitations of conventional CAD systems that rely on singular data modalities. By incorporating 46 genomic predictors, including frequently mutated genes and overexpressed miRNAs in thyroid cancer, the system delivers a novel and more accurate diagnostic approach. Furthermore, this comprehensive strategy extends existing CAD approaches by integrating ultrasound imaging and molecular data within a unified, modular diagnostic framework.

In the context of recent multimodal thyroid cancer detection studies, which increasingly combine ultrasound imaging with molecular testing or additional clinical variables to improve risk stratification and reduce unnecessary procedures, the proposed system follows a complementary design philosophy. While many existing approaches focus on benign versus malignant classification within preselected nodule cohorts, the present framework adopts a modular two-stage strategy, in which ultrasound-based screening is followed by molecular malignancy risk estimation when a nodule is detected. As a result, the system aligns with contemporary diagnostic workflows and addresses similar clinical objectives. Nevertheless, differences in study design, endpoints, and validation strategies across the literature limit direct metric-to-metric comparison, and the present results should therefore be interpreted within this broader methodological context.

Despite its promising outcomes, this study also presents several limitations that pave the way for future improvements. The ultrasound dataset used for training the CNN module was modest in size, and expanding it with larger, more diverse cohorts would further strengthen the generalizability of the image-based model. With respect to the molecular analysis component, the use of a GAN-based synthetic genomic dataset enabled systematic exploration of a large panel of genetic and miRNA predictors but also introduces inherent constraints. Synthetic data may not fully capture the complex distributions of real patient-derived genomic profiles, leading to potential distributional drift or the learning of dataset-specific artifacts. In addition, the inclusion of a large number of genomic and miRNA features raises the risk of feature dependencies and multicollinearity, as well as sensitivity to synthetic noise introduced during data generation. Although these effects were mitigated at the modeling stage through bounded input encoding, normalized feature weighting, and evaluation on independent training and testing sets, these measures do not replace validation on real clinical genomic data. Accordingly, the molecular module should be interpreted as a proof-of-concept, and external validation on real-world cohorts remains a necessary step to assess robustness and clinical relevance. Finally, although the two diagnostic modules can be used independently or in combination, broader prospective evaluations would be beneficial to assess their performance and usability in routine clinical settings.

Besides addressing the presented limitations, future developments will focus on refining the system to predict cancer progression, addressing the challenge of overdiagnosis in thyroid cancer. By correlating ultrasound image features with genomic markers, the system aims to stratify cancer risk and guide clinicians in tailoring patient management strategies.

## Figures and Tables

**Table 1:** Effect of different data augmentation techniques on the accuracy of ALEXNET.

Augmentation Technique	Model Accuracy [%]
No augmentation	78.4%
Random rotations (±20°)	81.8%
Random translations (±15 pixels)	82.6%
CLAHE	87.91%
Gaussian noise	90.48%

**Table 2:** Performance measures of the trained networks.

Neural Network	Accuracy [%]	Sensitivity [%]	Specificity [%]	Training Time [min s]
ALEXNET	90.48%	100%	53.84%	3 min 32 s
DARKNET-19	77.78%	98%	64.18%	6 min 08 s
VGG-16	88.89%	88%	92.3%	10 min 45 s
VGG-19	93.65%	100%	69.23%	15 min 25 s
RESNET-50	92.06%	100%	61.53%	18 min 46 s

## Data Availability

Data is unavailable due to privacy restrictions.
